# Reduced plasma levels of GM-CSF is a common feature of *Schistosoma mansoni*-infected school-aged children

**DOI:** 10.3389/fimmu.2025.1474575

**Published:** 2025-02-28

**Authors:** Severin Donald Kamdem, Leonel Meyo Kamguia, Alim Oumarou, Bernard Marie Zambo Bitye, Katie Lennard, Frank Brombacher, Thomas Spangenberg, Claudia Demarta-Gatsi, Justin Komguep Nono

**Affiliations:** ^1^ Unit of Immunobiology and Helminth Infections, Laboratory of Molecular Biology and Biotechnology, Institute of Medical Research and Medicinal Plant Studies, Ministry of Scientific Research and Innovation, Yaoundé, Cameroon; ^2^ Division of Microbiology and Immunology, Department of Pathology, University of Utah, Salt Lake City, UT, United States; ^3^ Faculty of Health Sciences, Protestant University Institute of Yaoundé, Yaoundé, Cameroon; ^4^ Ecole Doctorale Regionale (EDR) d’Afrique Centrale en Infectiologie Tropicale, Université des Sciences et Techniques de Masuku (USTM), Franceville, Gabon; ^5^ District Hospital of Mfou, Ministry of Public Health, Yaoundé, Cameroon; ^6^ Department of Integrated Biomedical Sciences, Division of Chemical and Systems Biology, Faculty of Health Sciences, University of Cape Town, Cape Town, South Africa; ^7^ Cape Town Component, International Centre for Genetic Engineering and Biotechnology, Cape Town, South Africa; ^8^ Immunology of Infectious Diseases Unit, South African Medical Research Centre, Cape Town, South Africa; ^9^ Wellcome Centre for Infectious Diseases Research in Africa, Institute of Infectious Diseases and Molecular Medicine (IDM), University of Cape Town, Cape Town, South Africa; ^10^ Global Health R&D of Merck Healthcare, Ares Trading S.A., (a subsidiary of Merck KGaA, Darmstadt, Germany), Eysins, Switzerland; ^11^ Division of Immunology, Faculty of Health Sciences, University of Cape Town, Cape Town, South Africa

**Keywords:** schistosomiasis, biomarker, GM-CSF, adjunct diagnostic, cytokine

## Abstract

**Background:**

Currently available schistosomiasis diagnostic and monitoring tools are limited, and the development of novel technologies is necessary to enhance disease diagnostic and surveillance by supporting elimination efforts. Novel disease-specific biomarkers can facilitate the development of these technologies. Through the comparison of parasite burden and host factors, we assessed whether host plasma cytokines could be used as robust biomarkers for intestinal schistosomiasis and associated pathology in school-aged children (SAC) living in endemic areas.

**Methods:**

Levels of host plasma cytokines were measured in SAC from a low-to-moderate burden region five months deworming with praziquantel, using Luminex assay for exploration analysis and ELISA for validation.

**Results:**

The concentration of GM-CSF, IL-2, and VEGF in plasma was significantly lower in schistosome-infected compared to non-infected children, as determined by Luminex assay. Further evaluation by ELISA revealed a negative correlation between GM-CSF plasma levels, but not those of IL-2 or VEGF, and *S. mansoni* egg burdens in infected individuals. Common coinfections in the study area such as geohelminths, hepatitis or malaria failed to alter plasma GM-CSF levels arguing in favor of a potential specific effect of *S. mansoni* infection on this cytokine. Receiver operating characteristic analysis confirmed GM-CSF as an acceptable predictive marker of *S. mansoni* infection, with an area under the curve (AUC) of 75%. Finally, the adjunct use of plasmatic GM-CSF thresholds for screening *S. mansoni* at-risk children and identify *S. mansoni*-infected ones increased the sensitivity of a single Kato-Katz test by averagely 15%.

**Conclusions:**

Our findings highlight the potential of using plasma GM-CSF levels to biomark *S. mansoni* infection and improve the sensitivity of single Kato-Katz based diagnostic for low- to-moderate burden infections.

## Introduction

Endemic in 78 countries, schistosomiasis remains the second greatest parasitic infection in terms of public health impact worldwide after Malaria (1). The latest figures indicate that approximately 780 million people are at risk of infection, with over 250 million individuals infected with *Schistosoma* spp (2).despite effective mass drug administration (MDA) of praziquantel (PZQ). The three main human schistosome species (*S. mansoni*, *S. haematobium*, and *S. japonicum*) evolve through three distinct stages of infection: acute, established active, and late chronic (3). While acute disease usually occurs in travelers, established active and late chronic schistosomiasis are mainly associated with long-term exposure to schistosomes in endemic areas (3). The severity of clinical symptoms can vary depending on the species of *Schistosoma* involved, the parasites number, the duration of the infection and host’s immune response to parasite eggs sequestered in tissues (4–6). Eggs trapped in the vasculature of the liver, intestines, bladder, or genital organs elicit host immune responses resulting in eosinophilic inflammatory and granulomatous reactions, which are progressively replaced by fibrotic (7), cancer (8) possibly leading to death (9).

Early detection and treatment are essential to prevent severe complications and reduce morbidity associated with the disease. Currently, the Kato-Katz (KK) thick stool smear technique (relying on the detection of eggs in stool) and the urine filtration method (relying on the detection of eggs in urine) through microscopical readout are the gold standard techniques for the diagnosis of intestinal *S. mansoni/S. japonicum* and urinary *S. haematobium* infections, respectively, in endemic areas. For *S. mansoni* infections, the KK technique provides a quantitative but indirect measure of infection intensity. It also helps to monitor the effectiveness of MDA programs but it is important to note its limitations. The main drawback is its underestimation of the true incidence/burden of schistosomiasis. Specifically, the dependence of the KK technique on eggs excretion is not reliable. Over time, egg excretion is a stochastic rather than deterministic process. As a consequence, the use of the KK technique to monitor the infection progression will result in underestimation thus limited sensitivity, especially in low-burden areas (10). A considerable improvement has been achieved with the recent development of parasite antigen detection tests such as circulating anodic antigen (CAA) and circulating cathodic antigen (CCA) which can detect parasite excretions/secretions in serum or urine at very low levels, which are indicative of infection (11, 12). However, these techniques can lead to false positive results as non-infected and pre-exposed (*in utero*) children from infected mother could display a positive CCA or CAA test (13–15). Additionally, previously infected people that have cleared the infection following treatment, could still be positive for the CCA or CAA tests (14). In contrast, polymerase chain reaction (PCR) is a more robust technic which detects the parasites nucleic acid, a proxy of the presence of the parasite. However, the technic is not cost effective and difficult to implement in remote area (16). Beyond diagnostic of infections, the KK, antigen and nucleic acid based diagnostic techniques are also limited to ensure a reliable assessment of the disease progression in infected hosts (4). This is in part due to the fact that we are now at the post-chemotherapy stage in most endemic countries. At this stage, prevalences are dropping (17) and thus parasite and actual pathognomonic parasitic products (antigens, nucleic acids) are not usually present in regularly treated hosts (instances of recent clearance). Additionally, with the accumulating evidence of poor linearity between infection burden (based on excreted egg burden) and consequent pathology during schistosomiasis (4, 18), it becomes critical in this post-MDA context to employ a combination of approaches. A mix of clinical assessment, unequivocal diagnostic tools, non-invasive imaging studies such as abdominal ultrasounds, and, when warranted, tissue biopsy should enable a more accurate diagnosis and grading of the disease. Unfortunately, such technology- and expertise-demanding procedures constitute logistical obstacles. This prevents the deployment and implementation of easy-to-use advanced diagnostic and morbidity monitoring tools in endemic rural areas further stressing the need for more innovative and complementary schistosomiasis diagnostic and monitoring tools (19).

Interestingly, the literature increasingly highlights host cytokine profiles as being recurrently altered by parasites in a distinctive manner (20). Illustratively, Ondigo et al. observed a noteworthy increase in soluble triggering receptor expressed on myeloid (sTREM) levels in children infected with schistosomiasis, a rise correlated not only with the onset of infection but also with subsequent egg burden (21). Furthermore, our group identified a negative correlation between *S. mansoni* infection and plasma interleukin (IL)-33 levels, with reduction closely reflecting increases in egg burden (22). Additionally, cytokines were reported to play a pivotal role in the onset or progression of liver fibrosis following hepatosplenic schistosomiasis (20). It is therefore clear that evidence gradually accumulate to define a comprehensive picture for a consensus host cytokine profile that might associate with schistosomiasis and/or cause a subset of chronically exposed patients to develop severe disease which is generally marked by tissue fibrosis.

In this study, we therefore attempted to better define a consensus host cytokine profile that might be pathognomonic of schistosomiasis and associated sequelae at the post MDA era. We probed systemically host plasma cytokine differences between schistosomiasis-infected and/or affected and non-infected children and evaluated the correlation between plasma cytokine levels, the likelihood of infection and the parasite burdens upon infection.

## Materials and methods

### Ethics statement

Ethical approval was obtained from the Cameroon National Ethics committee for Human Health Research (CNERSH) (No2018/02/976/CE/CNERSH/SP; No2021/12/1417/CE/CNERSH/SP, No2022/12/1505/CE/CNERSH/SP and No2024/02/639/CE/CNERSH/SP). Administrative authorizations for research were delivered by the Division of Health Operations Research of the Ministry of Public Health of Cameroon AAR No631-12.18 and the Ministry of Basic Education in Yaoundé, Cameroon. Additionally, authorizations were gathered from the Sub-Divisional Officer, the Chief-physicians, schools Directors and village leaders all in the Bokito subdivision where the study was conducted in five schools. Subsequently, written informed consent was obtained from all school children and their legal guardians. All data obtained were recorded in a questionnaire and treated anonymously. At the end of the study, all school children from the five represented schools within the study area were treated with PZQ, irrespective of their parasitological status.

### Study site and participants

**Figure 1:**
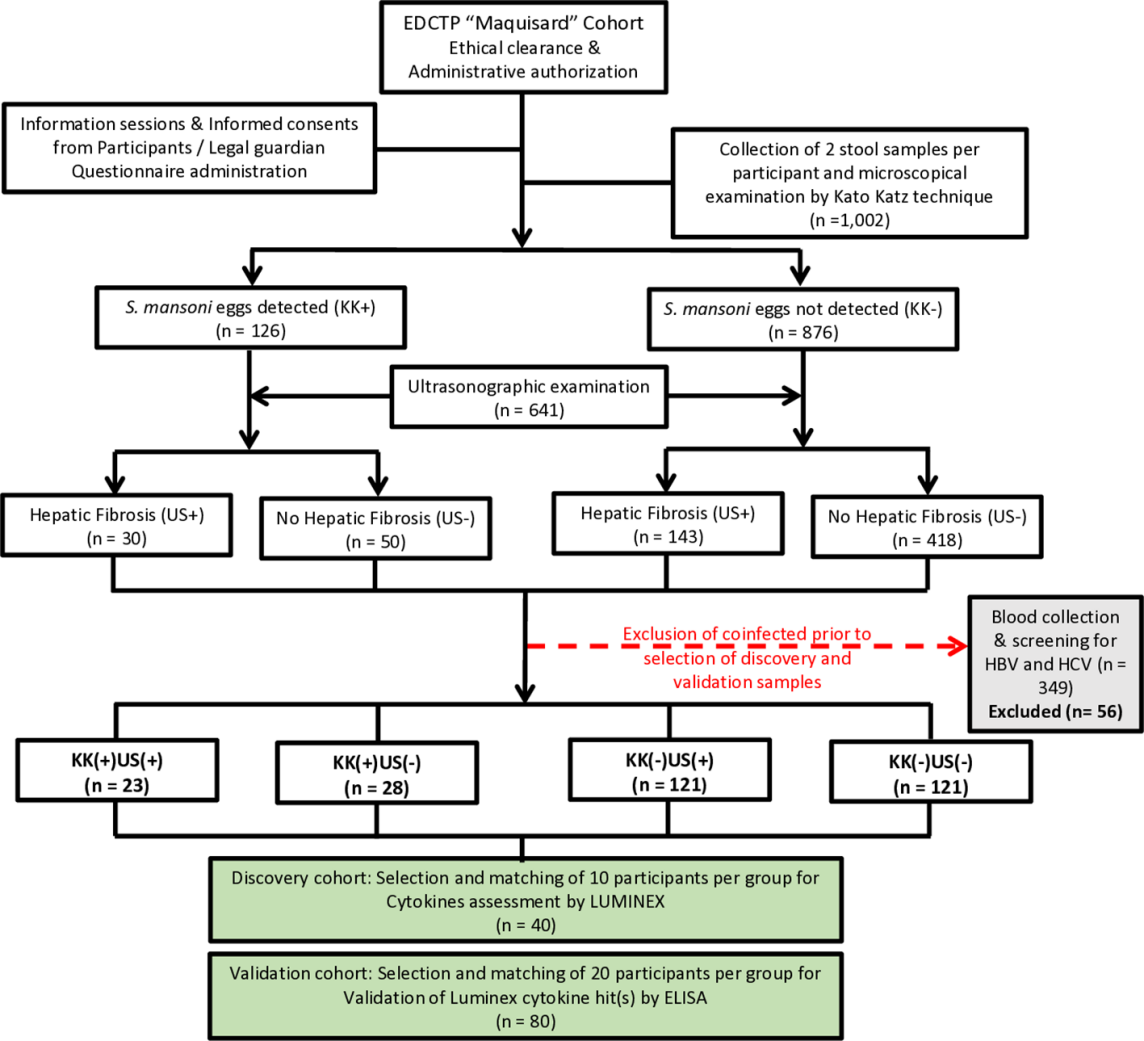
Flow diagram describing the enrolment strategy, examination process and designing of participant groups for the discovery and validation cohorts. For the selection of participants for the Luminex experiment (n=40) and enzyme-linked immunosorbent assay (ELISA) (n=80), infection was defined as the presence of *S. mansoni* eggs in the participant’s stool, as observed by KK technique; Liver fibrosis was defined using the liver image pattern (LIP) score. No hepatic fibrosis: Final score of 0, consisting of participants with only LIP A. Hepatic fibrosis: LIP score ≥ 2/(LIP C – LIP E). Participants were grouped into four groups based on the presence or absence of eggs in the stool (KK+ versus KK-) and the presence or absence of conclusive signs of liver fibrosis (US+ versus US-). Excluded participants: Degraded samples (n =13) or participants positive for HBV and/or HCV (n=43). This resulted in 4 phenotypic groups of patients, i.e., KK-US-, KK-US+, KK+US- and KK+US+. KK: Kato Katz; US: Ultrasonographic examination; (+): Positives; (-): Negatives. All participants with available blood samples were considered for further analyses and screened for concomitant infections such as geohelminths in stool samples, hepatitis B or C in blood and malaria thick smear from blood samples. All participants with another reported infection of the above was excluded from the further analyses to strictly enable *S. mansoni* mono-infections to be probed comparatively against non-infected controls. Two cohorts were constituted from the retained participants to perform first a discovery run of 10 participants per group (total, n = 40). Plasma samples selected for the discovery run were tested by Luminex for 27 cytokines to assess differential expression between groups with different infection and/or liver fibrosis statuses. The validation run was then performed with another 20 age-, gender-, BMI (body mass index)- and FCW (frequency of contact with infested water)- matched participants per group (total, n = 80) by cytokine-specific ELISA for confirmation of the biomarking potential of any candidate cytokine deemed interesting from the discovery run results. *Maquisard* study: An EDCTP and UK Royal Society funded study on school children from rural Cameroon.

We conducted a cross-sectional study in five public schools surrounding the infested rivers in five different villages namely *Bongando, Ediolomo, Kedia, Yoro 1* and *Yoro 2* in the Bokito subdivision, located in the Centre region of Cameroon, persistently endemic for *S. mansoni* infection (5). All consenting school children who had resided in the endemic area for at least six months, exhibiting apparent good health (as per their clinical examination), and were attending one of the 5 public schools selected during the time of data collection (September to December 2018), were eligible for inclusion in this study. Clinical signs of anemia were the main criteria of non-inclusion. Subsequently, participants were screened for schistosomiasis infection and liver pathology development using KK and ultrasound, respectively. Based on these screening results, participants were classified into four groups, and two cohorts were constituted (**Figure 1**). The study was conducted five months after MDA of PZQ by the Cameroon National Program for the Control of Schistosomiasis and Soil transmitted Helminthiasis.

### Data collection

#### Sociodemographic data

Each school child was interviewed by a member of our team, assisted by the parent/legal guardian and the class teacher. The questionnaire recorded all demographic data. This same approach was replicated in all five villages.

#### Samples collection and storage

Whole blood samples were collected as per established protocols (23). Briefly, blood samples were drawn into heparinized tubes (BD Vacutainer^®^, GRACE Vacuum Tube) and transported on ice to the Cameroon National Public Health laboratory (NPHL) in Yaoundé. Upon arrival, the tubes were centrifugated at 4°C (Eppendorf AG, Germany), and the plasma (supernatant) was carefully separated from the cell pellet, aliquoted and stored at -80°C until further analyses. Two stool samples for the detection of eggs were collected from each participant within a span of 5 days (Kato1 at day 0 and Kato2 at day 5) into a 50 ml pre-labelled and codified screw-cap vial stool container. Subsequently, within three hours, collected stool samples from each of the five sites were transported in coolers filled with ice packs to the laboratory for parasitological analyses. This procedure was replicated over the course of two days of stool collection.

#### Ultrasonographic examination of participants’ abdomen

A bench was set up in a private room on site to ensure participant privacy. School children were installed on the bench and examined using a portable ultrasonography device (ultrasound scan Vivid E, General Electric, Medical System, China, CO., LTD, Ref 5198983, SN 78856WX2) equipped with a convex transducer (Shenzhen Mindray Bio-Medical Electron I CS CO., LTD, SN AGH86175750) with adjustable frequencies, operated by a radiologist. The procedure followed previously described protocols (24). Pathologic lesions associated with *S. mansoni* infection were identified and recorded in accordance with the World Health Organization (WHO) guidelines on the assessment and quantification of *S. mansoni* morbidity, as outlined elsewhere (25). Participants with LIP strictly categorized as A were deemed unlikely to have fibrosis (25) and thus considered negative for hepatic fibrosis, whereas those with LIP ranging from C to F were classified as presenting with *S. mansoni*-specific hepatic fibrosis (26). Additionally, the presence of starry sky, pipe stems, and echogenic patches were noted, and the image pattern (IP) scores meticulously determined, as previously reported (22). Illustrations of various LIPs were previously reported in supplementary (22). Printouts of all participants’ ultrasonograms were retained for later independent validation of the initial conclusions.

#### Parasitological examination of stool sample by Kato Katz technique

From each stool sample, two KK smears were prepared and independently examined for the detection and quantification of parasites eggs using optical microscopy (Leica Microsystems, DM2000, Germany) at 10X and 40X magnifications, following established protocols (27). KK smears were used to detect and quantify *S. mansoni*, *S. haematobium* eggs ectopic elimination and other geohelminths as previously described (27). The prepared KK smears were examined by two experienced laboratory technicians. The arithmetic mean of eggs counted from the readings (four KK smears per participant) was considered as the participant burden. Additionally, for quality control assessment, 20 randomly selected slides were re-examined by a third laboratory technician for comparison. The eggs counted for each sample were recorded as eggs per gram (EPG), while samples with zero eggs were recorded as negative. The number of *S. mansoni* eggs was counted, recorded, and multiplied by 24 to determine the number of eggs per gram of feces. Infection intensity was classified as low (1–99 EPG), moderate (100–399 EPG), or heavy (≥400 EPG) according to the WHO guidelines (28). No *S. haematobium* eggs ectopic elimination was recorded in participants stool. However, geohelminths eggs were recorded in 31 school children (14 cases of *Trichuris trichiura*; 10 cases of hookworms i.e. *Ancylostoma duodenale/Necator americanus* and 7 cases of *Ascaris lumbricoides* with no cases of *Strongyloides* spp). Geohelminths coinfections were recorded in 3 participants (1 case of *Ascaris lumbricoides* & *Ankylostoma duodenale* coinfection; 1 case of *Ascaris lumbricoides* & *Trichuris trichiura* coinfection and 1 case of *Ankylostoma duodenale* & *Trichuris trichiura* coinfection). Only 4 cases of geohelminths coinfection with *S. mansoni* were encountered.

#### Malaria diagnostic

Microscopy for Malaria diagnosis was used in our study to identify *Plasmodium* parasites. Malaria thick smears were prepared and analyzed as previously described (29). Briefly, whole blood was mixed, and a spot placed in the center of a slide. Using the edge of another clean slide, red blood cells (RBC) were lysed to release *Plasmodium* parasites, and caution was taken to have a uniform thick smear (not too thin or too thick as they don’t stain well). This action was strengthened when adding the Giemsa solution which contains water and therefore leads to lysis of cells. Prepared thick smears were air dried, stained with a freshly prepared Giemsa solution (10%) and analyzed using an optical microscope. Parasites were then counted in parallel with 200 white blood cells (WBC) and the total number of parasites was multiplied by 40 to have the number of parasites/µl of blood. The total number of parasites was multiplied by 40 as in absence of WBC count, the WHO recommend the use of 8000 WBC/µl of blood per participant (30). A sample was declared negative after having screened at least 100 microscopic fields without parasites.

#### Screening for hepatitis B & C viruses

Participants were screened for hepatitis B and C viruses using DiaSpot HBsAg and DiaSpot HCV Ab rapid diagnostic test strips (DIASPOT™ Diagnostic, Indonesia), respectively (31). All positive participants were excluded from the study and referred for adequate management to the overseeing health facility (District Medical Centre, Bokito).

### Luminex assay

Plasma samples were analyzed using the Luminex platform (Bio-Plex Pro Human Cytokine Grp I Panel 27-Plex, BIO-RAD Laboratories) to quantify the concentration of 27 cytokines including interleukin (IL)-1β, IL-1 receptor agonist (IL-1ra), IL-2, IL-4, IL5, IL-6, IL-7, IL-8, IL-9, IL-10, IL-12p70, IL-13, IL-15, IL-17A, Eotaxin, fibroblast growth factor (FGF)-basic, granulocyte-colony stimulating factor (G-CSF), granulocyte-macrophage colony-stimulating factor (GM-CSF), interferon-γ (IFN-γ), IFN-γ inducible protein-10 (IP-10), monocyte chemoattractant protein-1 (MCP-1), macrophage inflammatory protein-1α (MIP-1α), MIP-1β, platelet-derived growth factor-BB (PDGF-BB), RANTES, tumor necrosis factor-α (TNF-α) and vascular endothelial growth factor (VEGF) as per the manufacturer’s recommendation. A total of 40 samples (from 40 discovery cohort participants) were run in duplicate on the same plate to avoid inter-plate variation. All markers were measured undiluted on a Bio-Plex 200 System (BIO-RAD Laboratories). The instrument was calibrated using a Bio-Plex Calibration kit (BIO-RAD Laboratories) prior to data acquisition. Data analysis was performed using Bio-Plex Manager software (version 6.0, BIO-RAD Laboratories), and cytokines concentrations were extrapolated from standard curves using a 5-parameters logistic regression. Analyte concentrations below the lower limit of detection of the assay were reported as the midpoint between zero and the lowest measured concentration for each analyte.

### ELISA assay

Human GM-CSF ELISA kit (RayBiotech, Cat No ELH-GM-CSF), IL-2 Sandwich ELISA Kit (proteintech, Cat No KE00017) and VEGF Sandwich ELISA Kit (proteintech, Cat No KE00216) were used to evaluate cytokine concentrations in participants’ plasma samples as per the manufacturer’s protocol. A total of 80 plasma samples were assessed individually. For both assays, plasma samples were diluted at a 1:1 ratio using the assay diluent. All samples fell within the detection range. Standards were assessed under the same experimental conditions as plasma samples, and standard curves were used to determine the concentration of each participant.

### Statistical analysis

Statistics were performed using R studio and GraphPad Prism 8 (https://www.graphpad.com/). Descriptive statistics, including means with standard error of the mean, percentages, and frequencies, were used to summarize the data. Graphs were plotted using GraphPad Prism 8. Comparisons between two groups were performed using the unpaired Student’s t-test, while one-way Analysis of Variance (ANOVA) was employed for comparisons across more than two groups. Multivariate logistic regression models were carried out to assess the relationship between cytokines and a given condition (*S. mansoni* infection or hepatic fibrosis), with adjusted odds ratios (AOR), 95% confidence interval (CI) and *p*-values reported. Unsupervised hierarchical clustering of participants was conducted using the R package “ComplexHeatmap”, utilizing log10-transformed cytokine expression data (32) and the results were annotated with class labels for the participants. Receiver Operating Characteristic (ROC) analysis was performed using the R library “pROC” to determine the diagnosis potential of candidate biomarkers (33). For all analyses, a *p*-value < 0.05 was considered statistically significant, and the specific statistical test used is mentioned in the corresponding figure legend.

## Results

### Demographic characteristics of participants

A total of 1,002 SAC were enrolled (gender ratio M/F of 1.1) in this study and divided into two experimental cohorts: a discovery cohort (n = 40) and a validation cohort (n = 80) both with the same M/F gender ratio of 1.1 as summarized in **Figure 1**. Study population characteristics are provided in **Supplementary Table S1**.

All participants underwent parasitological examination using KK technique to distinguish between infected and non-infected participants. *S. mansoni* eggs were found in the excreta of 126 participants. Afterwards, ultrasonographic (US) examinations were conducted on 641 (gender ratio M/F of 1.1) consenting participants to differentiate those with liver fibrosis from those without. Overall, participants were distributed into four groups based on both their *S. mansoni* infection and US liver disease status.

### Description of cohort constitution

Participants positive for both *S. mansoni* eggs (EPG ≥ 1) and liver fibrosis (IP score ≥ 2/LIP C – F) were grouped as KK(+)US(+); those positive for *S. mansoni* eggs (EPG ≥ 1) but negative for liver fibrosis (IP score = 0/LIPA) were grouped as KK(+)US(-). Participants negative for *S. mansoni* eggs (EPG = 0) but exhibiting evidence of liver fibrosis (IP score ≥ 2/LIP C – F) were grouped as KK(-)US(+), while participants free of both *S. mansoni* eggs (EPG = 0) and liver fibrosis (IP score = 0/LIPA) were grouped as KK(-)US(-).

Group selections were made while ensuring matching for potential confounding factors across groups (**Supplementary Table S1**). Similarities were observed in terms of age, BMI, gender, length of residence, FCW when comparing discovery and the validation (**Supplementary Table S1**).

Factors were selected based on their recognized potential risk for associating with schistosomiasis infection and disease i.e. age, gender, BMI and FCW. The matching of these risk factors across groups aimed to mitigate the biases they might cause when studying the role of cytokines during schistosomiasis infection and disease.

### Variation in SAC plasma cytokine levels: discovery cohort

**Figure 2:**
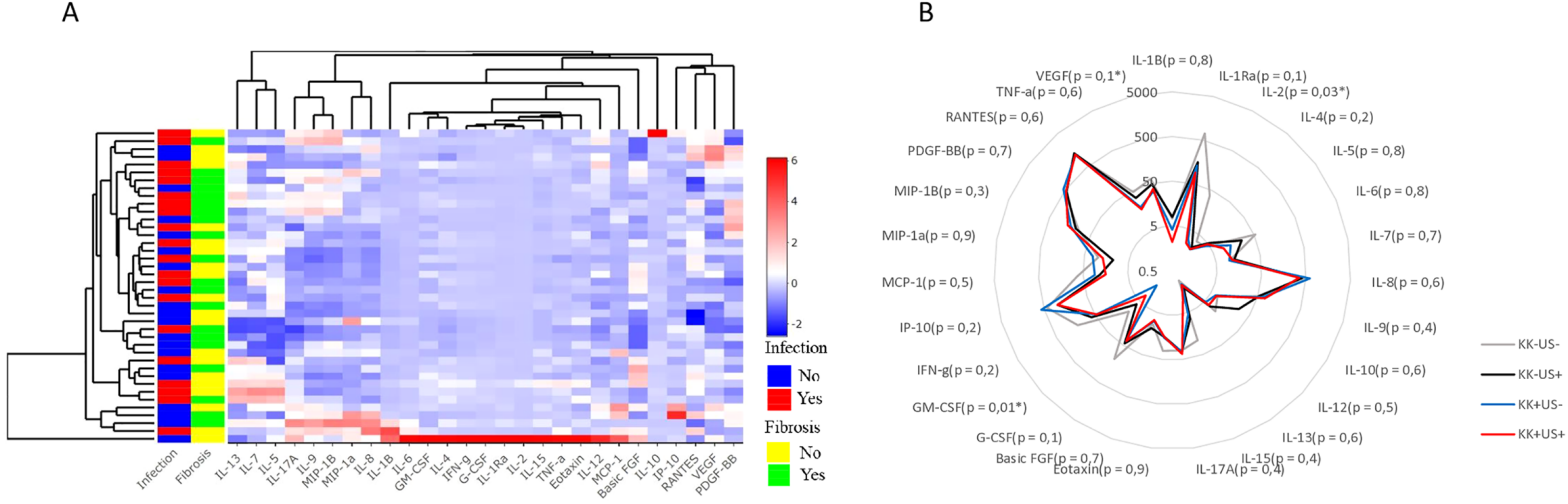
Cytokines profiles across participants infected with *S. mansoni* and/or hepatic fibrosis from the discovery run **(A)**. Heatmap after unsupervised hierarchical clustering based on cytokine expression similarity and showing the variation of the 27 cytokines expression in individual participant and per clusters. The R package “ComplexHeatmap” was used to generate the heatmap. **(B)** Radar plot showing the variation of the 27 cytokine’ expressions between the four groups of participants from the discovery run with indicated p-value of multiple comparison. For statistical comparison, using graph pad prism, Kruskal-Wallis test followed by Dunn test was performed to assess significant differences between 4 groups. (n = 40).

We profiled and quantified a panel of 27 human pro- and anti-inflammatory cytokines in plasma samples from 40 participants in the discovery cohort (10 per group) using a multiplex-based assay. No cytokines were exclusively present or completely absent from any of the samples tested. Preliminary analysis of the data by unsupervised hierarchical clustering used to visualize the variation in cytokine concentrations, failed to reveal major differences in cytokine expression across groups (**Figure 2A**). Furthermore, visualization of principal component analysis (PCA, **Supplementary Figure S1**) and radar plot (**Figure 2B**) confirmed a minimal variation in cytokine expression across the four groups.

Bar graphs for all the tested 27 cytokines revealed an apparent shift between groups for GM-CSF, IL-2 and VEGF (indicative but mostly literature supported, (34), as suggested by **Supplementary Figure S2**.

To determine whether these cytokines formed unique profiles for each group, multiple comparison were performed, revealing potential differences among groups for GM-CSF, IL-2, and VEGF (**Figures 2B**). While the other cytokines, did not differ significantly between clinical groups.

### Variations of GM-CSF, IL-2 and VEGF expression across groups: validation cohort

**Figure 3:**
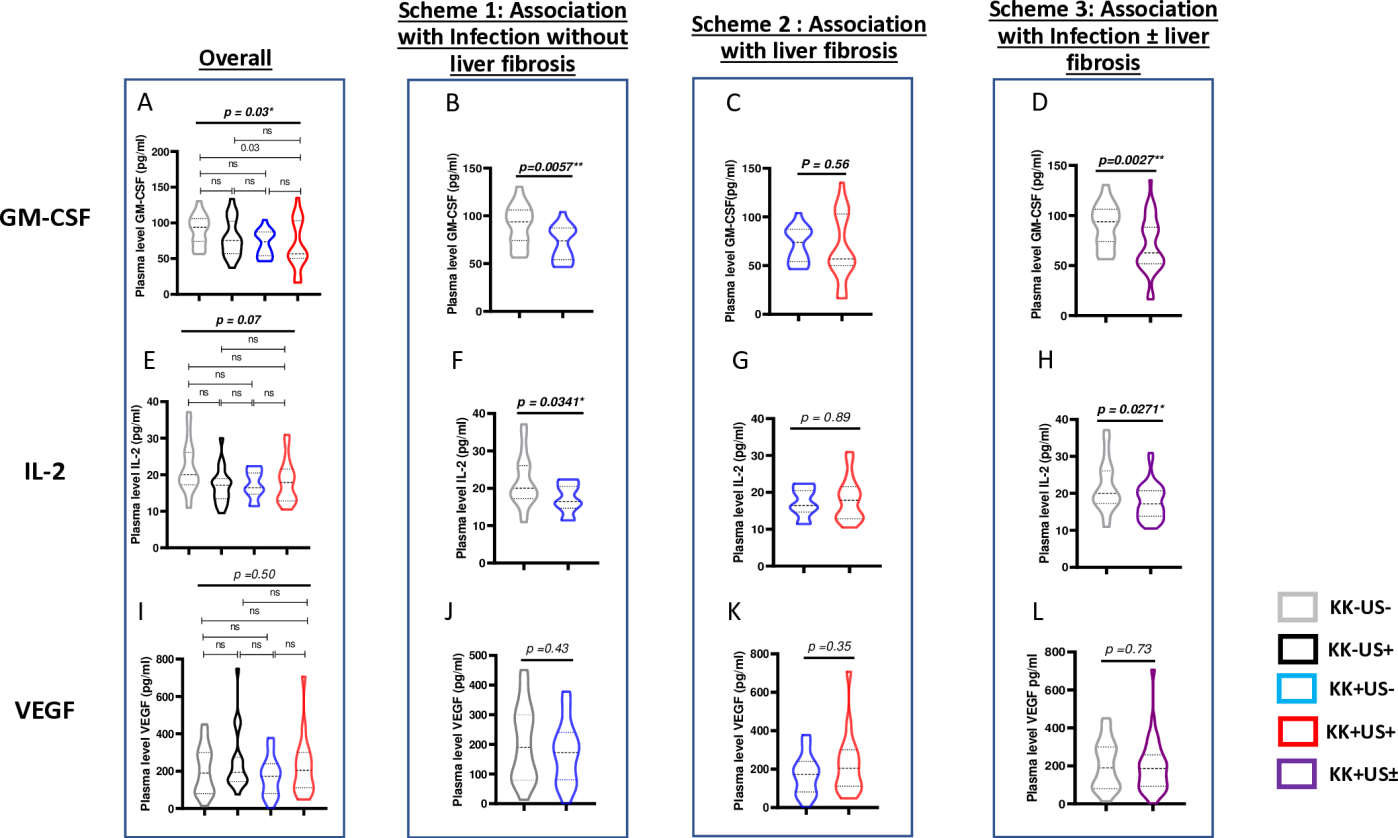
Differential cytokine expression during *S.mansoni* infection and/or liver fibrosis by ELISA across validation run samples. Four analytical univariate schemes are presented here i.e. overall: to unveil any suggestive (though not definitive) differences as a result of S mansoni infection and/or liver fibrosis. Scheme 1: to unveil any suggestive (though not definitive) differences as a result of *S. mansoni* infection before the onset of liver fibrosis. Scheme 2: to unveil any suggestive (though not definitive) differences as a result of liver fibrosis onset following *S. mansoni* infection. Scheme 3: to unveil any suggestive (though not definitive) differences as a result of any stage of *S. mansoni* infection. **(A)** <Comparison of plasma levels of GM-CSF across the 4 groups KK-US-, KK-US+, KK+US-, KK+US+. **(B)** Plasma levels of GM-CSF in KK-US- Vs KK+US-. **(C)** Plasma level of GM-CSF in KK+US- Vs KK+US+. **(D)** Plasma levels of GM-CSF in KK-US- versus KK-US-/+ (i.e. both KK+US- and KK+US+). **(E)** Comparison of plasma levels of IL-2 across the 4 groups KK-US-, KK-US+, KK+US-, KK+US+. **(F)** Plasma levels of IL-2 in KK-US- Vs KK+US-. **(G)** Plasma level of IL-2 in KK+US- Vs KK+US+. **(H)** Plasma levels of IL-2 in KK-US- versus KK-US-/+ (i.e. both KK+US- and KK+US+). **(I)** Comparison of plasma levels of VEGF across the 4 groups KK-US-, KK-US+, KK+US-, KK+US+. **(J)** Plasma levels of VEGF in KK-US- Vs KK+US-. **(K)** Plasma level of VEGF in KK+US- Vs KK+US+. **(L)** Plasma levels of VEGF in KK-US- versus KK-US-/+(i.e. both KK+US- and KK+US+). For statistical comparison, using graph pad prism, Kruskal-Wallis test followed by Dunn test was performed to assess significant differences between the 4 groups **(A, E, I)**. Mann Whitney U test was used to perform preliminary univariate comparison between groups in schemes 1,2,3. GM-CSF, Granulocyte Monocyte Colony Stimulating Factor; IL, Interleukin; VEGF, Vascular endothelial growth factor; KK-US-, negative for both *S. mansoni* eggs and hepatic fibrosis; KK-US+, negative for *S. mansoni* eggs and positive for hepatic fibrosis; KK+US-, positive for *S. mansoni* eggs and negative for hepatic fibrosis; KK+US+, positive for both *S. mansoni* eggs and hepatic fibrosis. ns= not significant; *=p<0.05; **=p<0.01.

A validation cohort of 80 samples (20 per group) was screened independently to confirm the findings obtained in the discovery cohort. More sensitive cytokine-specific ELISA assays were performed to assess whether plasma concentrations of GM-CSF, IL-2 and/or VEGF could be considered as potential candidate biomarkers of schistosomiasis infection and/or liver fibrosis (**Figures 3A, E, I**).

Three schemes of comparison (**Figure 3**) were used to assess any univariate (suggestive, but not definitive) variation of cytokine expression in plasma during schistosomiasis infection and pathogenesis and therefore ascribed any observed potential to either an association with infection and/or liver fibrosis:

- Scheme 1 comparison showed that plasma levels of GM-CSF (**Figure 3B**) and IL-2 (**Figure 3F**) were significantly lower in KK(+)US(-) group when compared to levels in the control group KK(-)US(-) (p = 0.005 and p = 0.03 respectively) revealing a likely reduction of GM-CSF plasma levels that associates with *S. mansoni* infection.
- While, scheme 2 comparison showed that plasma levels of GM-CSF and IL-2 did not vary significantly between *S. mansoni*-infected children with or without liver fibrosis groups (**Figures 3C, G, K**) suggesting a lack of association of the plasma levels of these cytokines with the onset/progression of liver fibrosis during *S. mansoni* infection.
- Finally, GM-CSF and IL-2 plasma levels were also significantly lower in the infected group (irrespective of the liver status) when compared to the control group KK(-)US(-) in scheme 3 (p = 0.003 and p = 0.03; **Figures 3D, H**) further arguing in favor of a reduced levels of these cytokines in children infected by *S. mansoni*. Of note, however, no significant difference was observed for VEGF plasma levels in all three comparison schemes (**Figures 3J, L**).

To unequivocally assess the statistical robustness of the observed suggestive influences of *S. mansoni* infection on the candidate cytokine biomarkers confirmed in the above univariate analyses i.e. GM-CSF and IL-2 as being negatively associated with *S. mansoni* infection (**Figure 3**), we next proceeded to perform a multivariate logistic regression analysis of risk factors of *S. mansoni* infection in our study assessing the odds of our candidate cytokines in altering the likelihood of *S. mansoni* infection independently from age, gender, BMI and FCW (**Table 1**). Only GM-CSF levels appeared to significantly present with a likelihood to negatively associate with *S. mansoni* infection (aOR=0.9; p=0.02) whereas neither IL-2 nor VEGF plasma levels did show such odds (p=0.6 and 0.3, respectively). Notably, an elevated but non-significant odd of being *S. mansoni* infected with higher FCW was also reported (aOR=44.7; p=0.09 from GM-CSF ELISA data) consolidating the known fact that increased exposure to infested water increases the likelihood of being infected.

**Table 1:** Multivariate logistic regression to assess the influence of candidate cytokine levels on the likelihood of being *S. mansoni* infected in our study population.

FACTORS	Adjusted odds ratio (aOR)	95% Confidence interval (CI)	P VALUE
GM-CSF			
GM-CSF	0.9055	0.8040 to 0.9696	0.0283
Age	1.444	0.4728 to 5.366	0.5333
Gender	0.3621	0.01921 to 4.869	0.4510
BMI	0.5872	0.09551 to 2.399	0.5013
FCW	44.73	1.824 to 18715	0.0999
IL2			
IL-2	0.9636	0.7989 to 1.158	0.6850
Age	0.5865	0.2254 to 1.303	0.2176
Gender	0.3191	0.02619 to 3.085	0.3361
BMI	2.128	0.7186 to 8.305	0.2029
FCW	1.890	0.5686 to 7.162	0.3110
VEGF			
VEGF	1.005	0.9960 to 1.014	0.3189
Age	0.5503	0.2003 to 1.250	0.1891
Gender	0.2209	0.01636 to 2.112	0.2113
BMI	2.247	0.7865 to 8.279	0.1632
FCW	1.573	0.4413 to 6.217	0.4885

After correcting for age, gender, BMI, FCW and testing adjusted odds ratios of GM-CSF, IL-2 and VEGF, only plasma levels of GM-CSF appear to significantly reduce the risk of being *S. mansoni-*infected.

### Plasma levels of GM-CSF correlates (negatively and) specifically with *S. mansoni* burden

**Figure 4:**
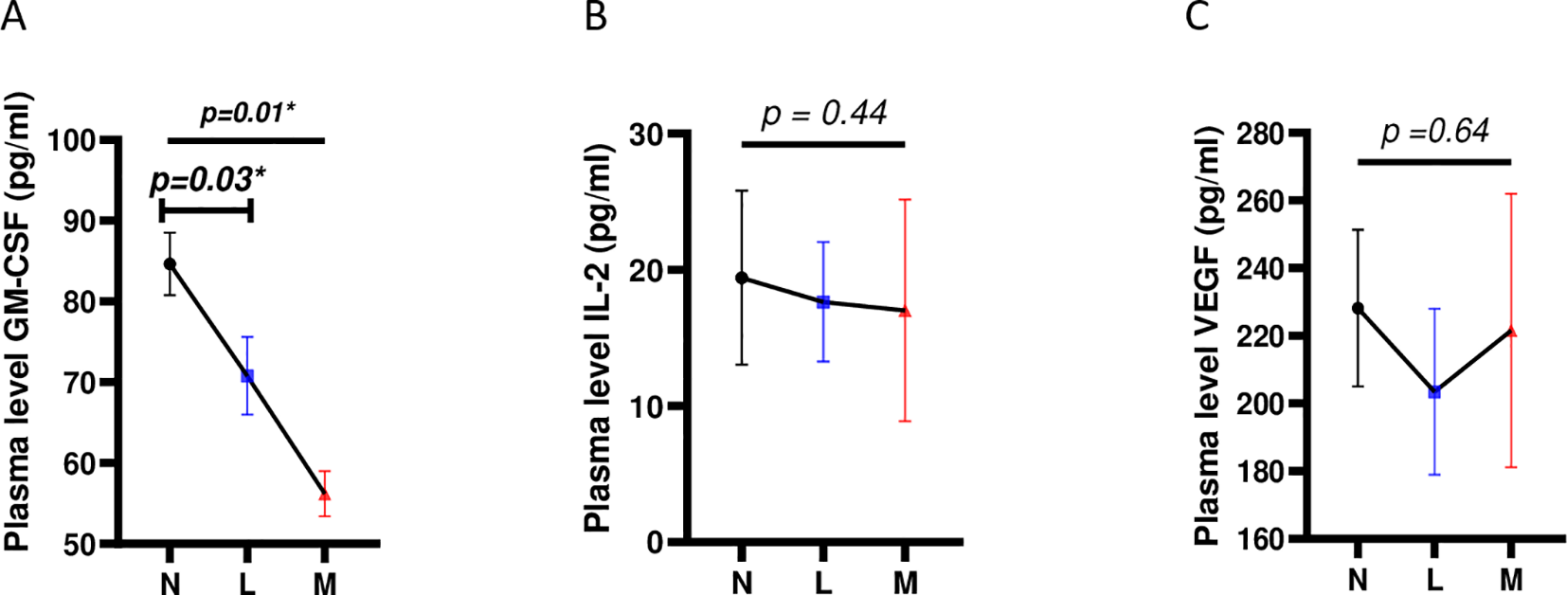
Correlation of levels of plasma cytokine candidate biomarkers with *S. mansoni* infection burden. **(A)** Correlation between plasma levels of GM-CSF and *S. mansoni* infection burden **(B)** Correlation between plasma levels of IL-2 and *S. mansoni* infection burden. **(C)** Correlation between plasma levels of VEGF and *S. mansoni* infection burden. For statistical comparison, using graph pad prism, Kruskal-Wallis test followed by Dunn test was performed to assess significant differences between the 3 egg burden groups. p< 0.05 was considered significant. GM-CSF, Granulocyte Monocyte Colony Stimulating Factor; IL, Interleukin; VEGF, Vascular Endothelial Growth Factor; N, Negative for *Sm* eggs; L, light infection (1–99 EPG); M, moderate infection (100–399 EPG); EPG, eggs per gram of stool. *=p<0.05.

An assessment of the relationship between plasma levels of GM-CSF, IL- 2, and VEGF and infection burden (egg load) was conducted. We classified participants’ burdens based on WHO guidelines, i.e. Light intensity of infections (L) or Moderate intensity of infections (M) (28). No cases of heavy intensity of infection (H) in our validation study were reported and participants with 0 egg per gram of stools were classified as nil (N). We noted that GM-CSF plasma levels significantly decreased when *S. mansoni* infection burden increased (p=0.01) (**Figure 4A**). In contrast, IL-2 and VEGF plasma levels were not associated with *S. mansoni* infection burden in any way (**Figures 4B, C**). This correlation of GM-CSF plasma levels with *S. mansoni* infection further supported our multivariate analysis where only plasma levels of GM-CSF, but not those of IL-2 of VEGF, presented with a significant association (p=0.03; aOR=0.90; CI=0.804-0.969; **Table 1**), though negative, with *S. mansoni* infection.

Coinfections are common in rural settings, such as our study site of rural Cameroon, increasing the likelihood that the observed reduction in GM-CSF may not be specific to *S. mansoni* infection and thus highlights a potential gap in our observations. This possibility is supported by reports of negative modulation of host GM-CSF production in response to some geohelminths. Our screening efforts during both discovery and validation phases (**Figure 1**), as well as an additional analysis of a subset of stored samples to assess the range of coinfections at our study site (**Supplementary Figure S3**), did not detect local cases of *S. stercoralis*. However, we did identify several lung-transiting geohelminths, such as *A. lumbricoides*, hookworms, *T. trichiura*, and blood-dwelling infectious agents like hepatitis B, hepatitis C, and malaria (**Supplementary Figure S3A**), which are commonly found in SAC exposed to *S. mansoni* in the area. Comprehensive analyses of GM-CSF levels in the plasma of individuals harboring these infections, both with and without *S. mansoni*, (**Supplementary Figures S3B, C**) did not reveal any alterations in GM-CSF levels due to these concomitant infections. This further strengthens the conclusion that the observed reduction in plasma GM-CSF levels in our study setting is specific to *S. mansoni* infection, even in the presence of widespread coinfections.

### Plasma levels of GM-CSF allow differentiation between *S. mansoni*-infected and non-infected participants

**Figure 5:**
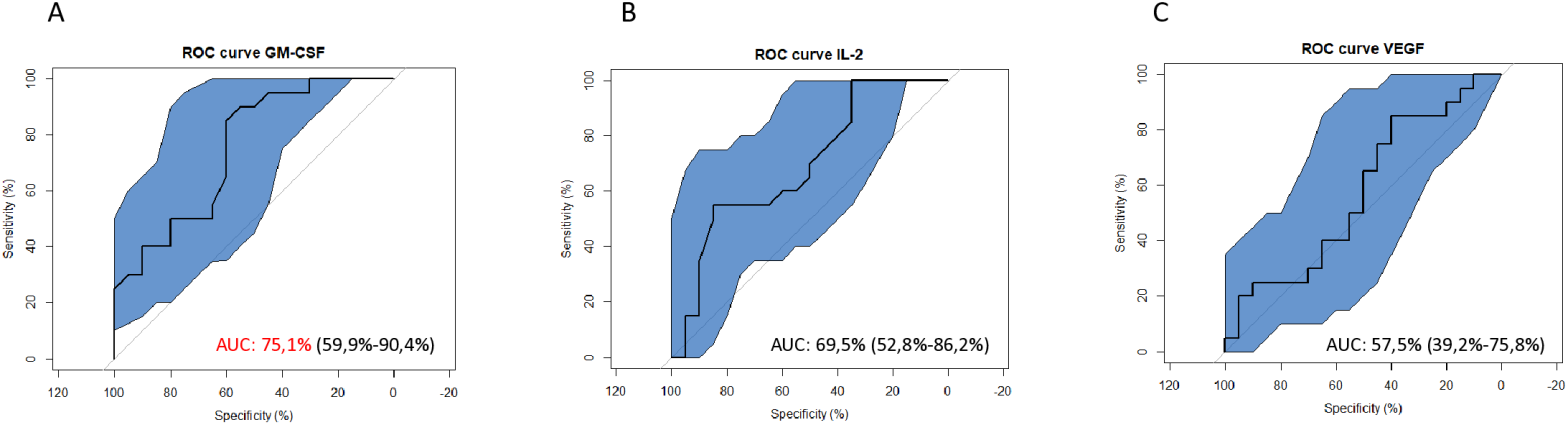
Receiver Operating Characteristic (ROC) curves to assess the diagnostic potential of *S. mansoni-*infected individuals using plasma levels of identified candidate cytokine biomarkers. **(A)** ROC curves showing the potential of GM-CSF in the diagnosis of *S. mansoni* infection. **(B)** ROC curves showing the potential of IL-2 in the diagnosis of *S. mansoni* infection. **(C)** ROC curves showing the potential of VEGF in the diagnosis of *S. mansoni* infection. GM-CSF, Granulocyte Monocyte Colony Stimulating Factor; IL, Interleukin; VEGF, Vascular Endothelial Growth Factor; AUC, Area Under the Curve; ROC, Receiving Operating Curve.

ROC curves were plotted to assess cytokine plasma levels as potential biomarkers for *S. mansoni* infection (**Figure 5**). Analysis of GM- CSF expression showed an AUC equal to 0.75 (95%CI=0.60-0.90; **Figure 5A**), which is considered valid for a predictive parameter (35) in discriminating here between control groups [KK(-)US(-)] and *S. mansoni* infected group without liver fibrosis [KK(+)US(-)]. Meanwhile, IL-2 and VEGF with AUCs of 0.57 (95%CI=0.53-0.86; **Figure 5B**) and 0.69 (95%CI=0.39-0.76; **Figure 5C**), respectively, performed less well as predictive parameters (35) in differentiating between non-infected KK(-)US(-) and *S. mansoni*-infected KK(+)US(-) groups.

**Figure 6:**
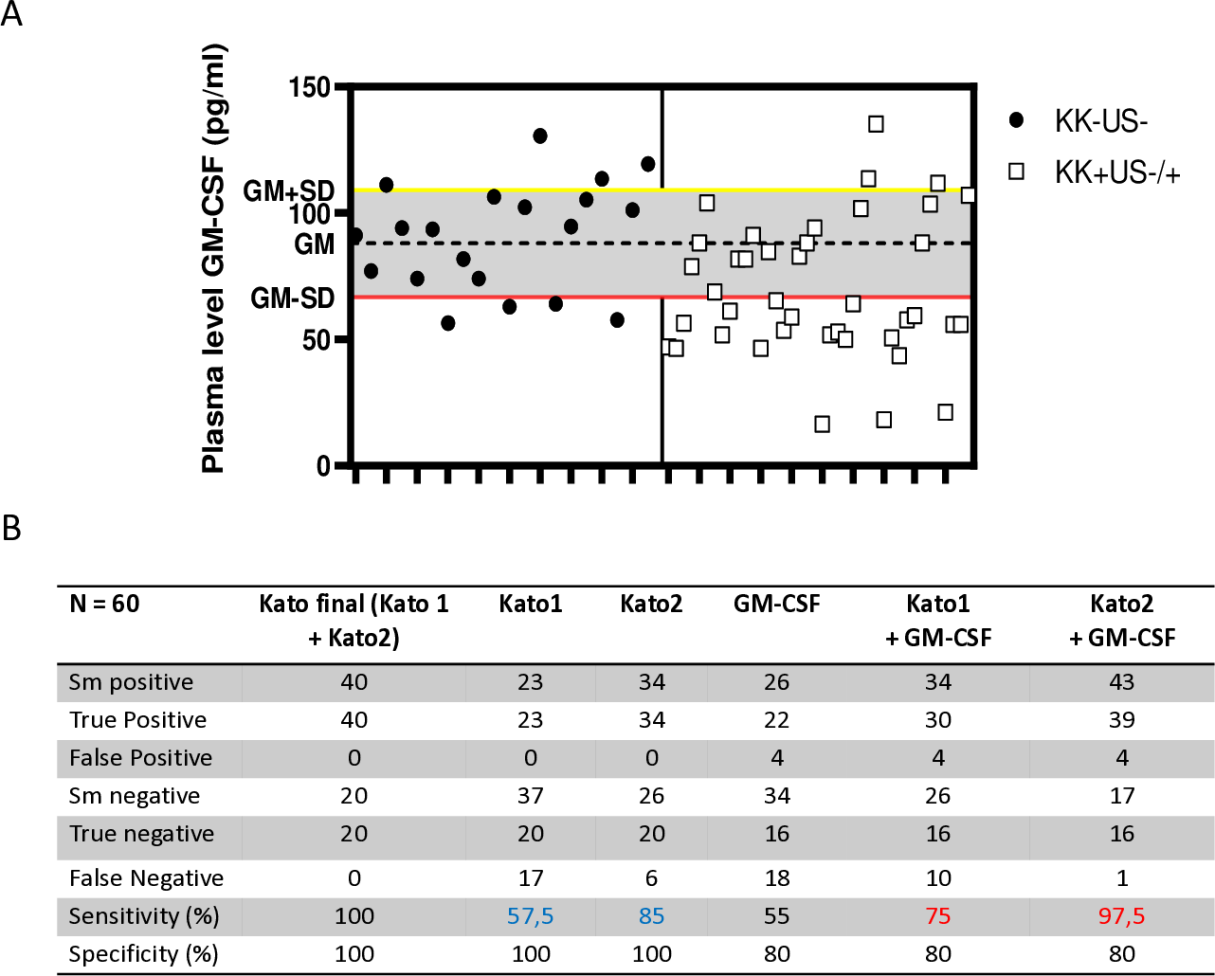
Adjunct diagnostic potential of plasma GM-CSF levels in better identifying *S. mansoni-infected* individuals after a single Kato-katz. **(A)** Plasma levels of GM-CSF threshold design for the separation of non-infected and *S. mansoni-*infected children. Plasma levels of GM-CSF were used as a diagnostic tool, with the geometric mean minus standard deviation (GM-SD) of non-infected individuals representing the lower cut-off (red line) separating non-infected (above) from infected individuals (below). **(B)** Summary table of the performance of each KK, plasma levels of GM-CSF, combination of single KK and GM-CSF-based identification versus our study gold standard (i.e. two repeated KK, Kato 1 and Kato 2, with each performed in duplicate). The sensitivity and specificity of the several diagnostic approaches were used against Kato final (the gold standard of mean of Kato1 and Kato2). Kato1 was performed after the first sample stool was collected, whereas Kato2 was performed after the second sample stool was collected from the same individual 5 days later. The sensitivity of the combination of plasma levels of GM-CSF with either Kato1 or Kato2 were also determined (in red) and compared to the original sensitivity of the single KK-based detection (in blue).

Based on these observations, the discriminative power of GM-CSF as potential candidate biomarker for *S. mansoni* infection was further assessed by comparing how plasma levels of this cytokine performed in comparison to KK for the identification of *S. mansoni*-infected children (**Figure 6**). Practically, the mean ± standard deviation of GM-CSF plasma levels from children in the reference group [KK(-)US(-)] was set as the baseline of GM-CSF levels and all tested GM-CSF plasma levels below this defined threshold were considered to be those of *S. mansoni*-infected individuals. All analyzed GM-CSF levels were therefore categorized as *S. mansoni* negative if falling below this threshold area whereas all GM-CSF plasma levels found at or above this threshold area were defined as non-infected (**Figure 6A**). In parallel, KK results from the screened children (performed at 2 time points i.e. Kato 1 and Kato 2) were used as reference with a combination of both KK used here as the study gold standard to determine sensitivity and specificity of all methods (**Figure 6B**).

When compared to KK performed on a single collection of stools at a given time point and analyzed in duplicates i.e. Kato1 or Kato2, plasma GM-CSF levels demonstrated lower sensitivity (55% *vs*. 57% and 85%) and lower specificity (80% *vs*. 100%). However, when combined to single KK readings, the use of plasma GM-CSF levels to identify *S. mansoni*- infected children consistently ameliorated the sensitivity (from 57% to 75% and from 85% to 97%) of any single KK but did not ameliorate the specificity (100% versus 80%). Notably, therefore, plasma GM-CSF-based identification of *S. mansoni*-infected children could ameliorate by 12-23% the sensitivity of a single KK.

## Discussion

Schistosomiasis control requires simpler and more effective tools that can be used for the mapping of infections and monitoring of disease. Such a need highlights the requirement to expand research into developing alternative, minimally invasive and logistically easy-to-use approaches (19). Notably, in response to infection, it is established that the host immune system modulates the production or inhibition of various cytokines, which in turn are instrumental in determining the outcome of infection and associated disease (6, 22).

In this study, we sought to determine which host plasma cytokines, based on their expression levels, might help distinguish between infected and non-infected SAC and/or monitor the presence of liver fibrosis in infected SAC from a well reported *S. mansoni*- endemic area in rural Cameroon i.e. the health area of Bokito (5). To achieve our objective, we meticulously screened participants with stringent clinical criteria to ensure that clinical phenotypes were as pure as possible. Our vision was to attribute any differences in cytokine expression predominantly, if not entirely, to the clinical traits that characterize SAC from schistosomiasis endemic areas namely the presence of *S. mansoni* eggs in the stool by KK and the US identification of liver lesions indicative of liver fibrosis. To limit bias from concomitant co-endemic diseases in the area, SAC were screened for malaria, hepatitis and STH for exclusion and parallel testing. Our screening resulted in the generation of four clinical groups of SAC namely i) without *S. mansoni* eggs in stool and with no signs of liver fibrosis i.e. KK- US-, ii) without *S. mansoni* eggs in stool and with liver fibrosis i.e. KK-US+, iii) with *S. mansoni* eggs in stool and with no signs of liver fibrosis i.e. KK+US- and iv) with *S. mansoni* eggs in stool and with liver fibrosis i.e. KK+US+. To further ensure unbiased comparability between the groups, SAC were selected to achieve similarity in mean age, gender distribution, mean BMI and mean FCW across groups. The resulting groups were further distributed into two cohorts, a first discovery cohort of 10 SAC per group for exploratory screening of plasma cytokines by high throughput Luminex assay of 27 cytokines and a second validation cohort of 20 SAC per group for confirmatory assessment by ELISA of candidate biomarking cytokines identified in the discovery run. Our selection proceedings strictly ensured that in both cohorts the safekeeping of all prerequisites listed above (unambiguous clinical classification, absence of concomitant infections, comparable mean ages, mean BMI, Gender ratio and mean FCW across groups) was upheld.

From the panel of 27 cytokines assessed for any cytokine differentially expressed between our 4 groups, whereas no cytokine showed an altered expression between groups that could be indicative of an association with the presence of liver fibrosis, we observed that GM-CSF, IL-2, and VEGF were significantly diminished in plasma samples obtained from *S. mansoni*-infected participants. These alterations suggest a potential dysregulation of immune responses in *S. mansoni*-infected individuals characterized by lower levels of key cytokines involved in steps of various immunological processes including innate immunity [GM-CSF, (36)], adaptive immunity [IL-2, (37)] and angiogenesis [VEGF, (38)]. It is important to note the limited spectrum of liver fibrosis cases reported, mostly characterized by mild cases. Consequently, screening for cytokine biomarkers in areas with more severe tissue fibrosis may yield different results, and this warrants further investigating. Regarding the observed changes associated with *S. mansoni* infection in our study population, more in-depth analyses, including correlation assessments between these cytokines and their ability to differentiate *S. mansoni* infection intensities (light, moderate, and heavy), revealed an inverse correlation between GM-CSF plasma levels and *S. mansoni* infection burden. This correlation, not observed with the other candidate biomarker cytokines, suggests that alterations in GM-CSF expression, specifically, known to contribute to maintaining immune homeostasis and promotes inflammation in helminthic infections (39), may uniquely reflect host immune response induced by exposure to *S. mansoni*. A biomarking potential of plasma levels of GM-CSF is therefore unprecedentedly suggested.

Consistent with our results, a recent study highlighted a negative correlation between the GM-CSF plasma levels and *S. stercoralis* infections (40). Additionally, GM-CSF is known to stimulate neutrophils, eosinophils, and monocytes/macrophages, enhancing gut barrier function, resistance to bacterial translocation, and enhancing tissue repair in the intestines (39).

Therefore, according to these reports, host GM-CSF may either play a crucial role in parasite control by stimulating host immune cells to inhibit parasite growth and development (41). Hence, the observed reduction of plasma levels of the cytokine could come as a result of over utilization for the protective response elicited in *S. mansoni*-infected hosts. In fact, several reports have demonstrated a robust myeloid activation following schistosomiasis, a process that is known to be centrally regulated by elevated GM-CSF levels (42). Various cell types, including granulocytes, epithelial cells, mesothelial cells, tumor cells, and in majority, fibroblasts, endothelial cells, monocytes/macrophages activated T and B cells produce GM-CSF (42) during schistosomiasis independently of the disease progression. The sequestration of those GM-CSF source cells, such as macrophages and fibroblasts, in the granuloma could explain this heavy solicitation hypothesis. Unfortunately, GM-CSF source cells were not monitored during our study, warranting further investigations in that regard to assess this hypothesis.

Another possible explanation for the reduced levels of plasmatic GM-CSF in *S. mansoni*-infected individuals could simply be parasite-driven impairment of GM-CSF production by host cells. Although not yet specifically described within *Schistosoma* spp. parasite immune-modulatory tools, reports strongly suggest the robust ability of schistosomes to impair T cell and macrophage functions (43), which are two central sources of GM-CSF during inflammation (42). Nevertheless, with no evidence clearly supporting the hypothesis of the parasite-driven reduction of GM-CSF levels, available literature instead reports on the enhancing role of GM-CSF in fostering the alternative activation of macrophages (44), a process critically sustained by the host to preserve its integrity and survival during schistosomiasis in the murine model (45). This further supports the theory of heavy solicitation of the cytokine at the tissue level to enable the host’s critical response against the parasite, resulting in reduced plasmatic levels (46). Collectively, whether GM-CSF supplementation might foster resistance to *S. mansoni* infection warrants further investigation. Importantly, our data, derived from a polyparisitized set of samples, suggests that the reduction in GM-CSF levels is not driven by any of the co-occurring infectious diseases such as geohelminths, hepatitis or malaria. This promising observation highlights the strong and specific potential of using plasma GM-CSF levels as a host biomarker of *S. mansoni* infections, though not liver fibrosis, in SAC. Given the urgency for alternative or improved diagnostic tools, this findings aligns with the recently launched 2021-2030 WHO roadmap for neglected tropical diseases (19), we proceeded to investigate the performance of host plasma GM-CSF as a practical discriminative tool for diagnosing *S. mansoni* infection. The rationale behind this investigation stems from the limitations of current diagnostic and monitoring methods in the global control strategy against schistosomiasis (47). Currently, two KK thick smears from a single stool sample are still commonly used by most national control programs for diagnosing *S. mansoni* infections to map areas for intervention and monitor impact of MDA of PZQ (48), despite their low sensitivity in low burden MDA-subjected areas (49). A seminal report has shown a sensitivity oscillating between 55%-90% for two KK thick smears from a single stool sample in an area subjected to repetitive MDA of PZQ (49), as is our study site. The common use of such a limited diagnostic/monitoring approach (single stool collection), clearly suboptimal, is however justified by the need to minimize the logistical cost in time and resources that might be required to collect more than a single stool sample at different time points. Our study screened SAC rather using four thick smears i.e. (two stool samples analyzed in duplicates) as gold standard, given the literature report of a sensitivity of 75%-95% for this combination of KK from 2 stool samples (49). Compared to such a ‘gold standard’, our work revealed the potential of complementing *S. mansoni*-infected SAC identification by plasma levels of GM-CSF to augment the sensitivity of 2KK from a single stool sample by up to 15%. Practically, when confirmed in multiple settings (i.e., longitudinally in larger and poly-parasitized cohorts), mapping and monitoring studies with only single stool samples collected could make use of plasma levels of GM-CSF, when applicable, to augment the sensitivity of their screening. This represents the glimpse of a useful opportunity for the 2021-2030 WHO NTD roadmap, more so as currently developed alternative diagnostic and monitoring tools as point of care (POC) tests i.e. POC-CCA, although promising and wonderful addition also presents issues of low sensitivity when applied to low burden infections (49). It will therefore be interesting to assess how the herein proposed combination with plasma levels of GM-CSF will affect the performance of such test in the diagnostic of schistosomiasis in low burden settings.

**Figure 7:**
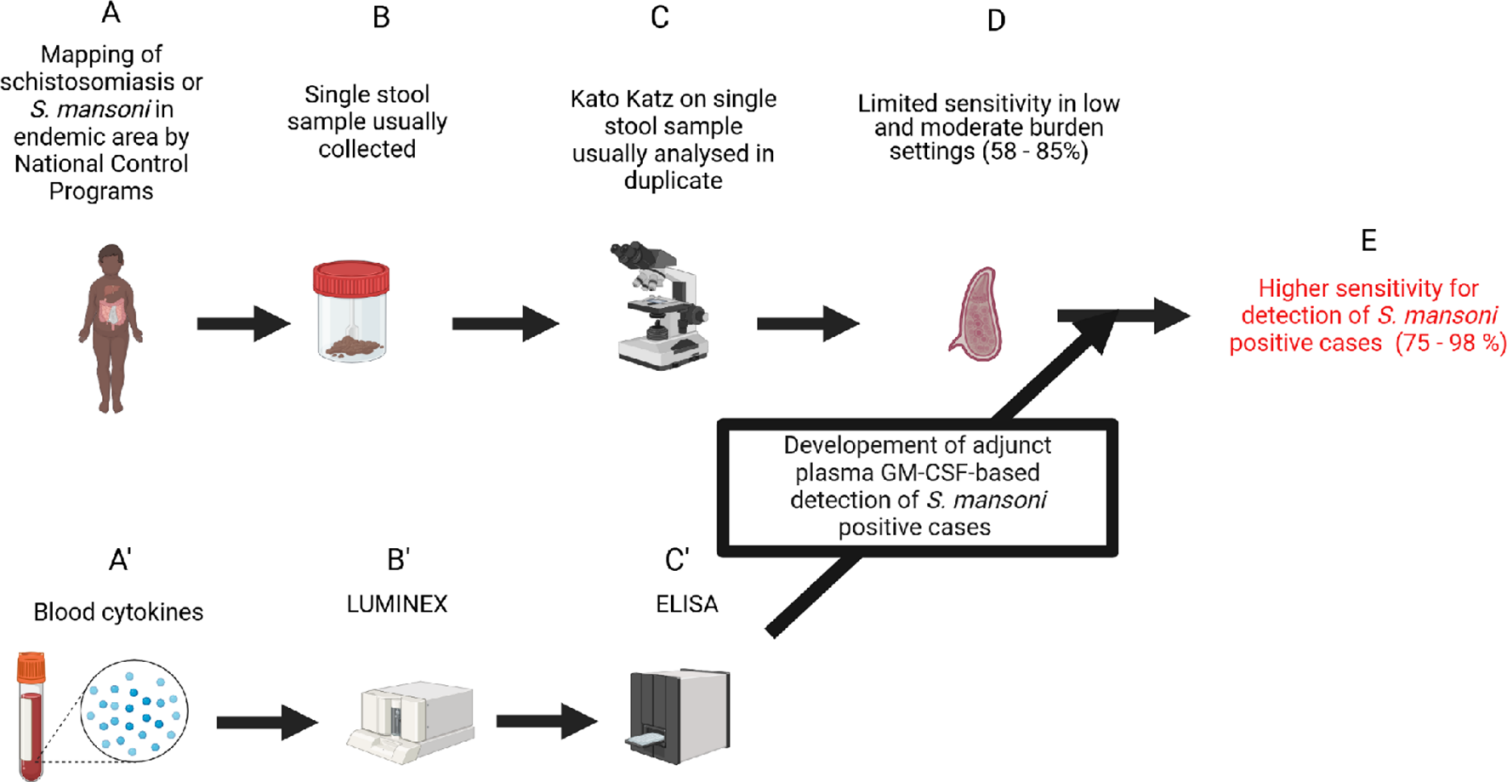
Study pathway to impact. Biorender.com software was used to design this workflow. **(A)** Detection of eggs in stools samples was used to diagnose schistosomiasis infection. **(B)** Kato Katz method is recommended for egg detection in stool samples and usually conducted on stool from a singlecollection day. **(C)** Analysis of stool from a single collection day by the KK method, even in duplicate, presents a low sensitivity in the diagnosis of schistosomiasis infection. **(A’)** Assessment of the role of host cytokine level changes during schistosomiasis. **(B’)** Large screening of host plasma cytokines using the Luminex method in a discovery run. **(C’)** Validation of cytokine candidates obtained from the discovery run after statistical analyses on other cohorts (with or without coinfections) using cytokine-specific ELISAs. **(D)** Identification of plasma GM-CSF as a robust plasma biomarker and use as adjunct diagnostic tool for the common single day stool-based KK achieving a 15% increase in sensitivity **(E)**.

In conclusion, we report, for the first time, a negative association between GM-CSF plasma levels and *S. mansoni* infection in SAC. The observed correlation between lower plasma GM-CSF levels and increasing *S. mansoni* egg burden underscores the potential significance of GM-CSF as a biomarker for infection and associated burden in endemic areas. Additionally, this reinforces its potential clinical significance in the management of schistosomiasis. The likelihood of integrating GM-CSF assessment into routine diagnostic and monitoring protocols unprecedentedly constitutes a prospect of ameliorated diagnostic (**Figure 7**), enhanced risk stratification, optimized treatment strategies, and improved patient outcomes in schistosomiasis endemic regions. Therefore, the confirmation of the biomarking potential of plasma GM-CSF levels for *S mansoni* infection in larger, longitudinal and more poly-parasitized cohorts is hereby suggested as a promising avenue for future investigations.

## Data Availability

The original contributions presented in the study are included in the article/**Supplementary Material**. Further inquiries can be directed to the corresponding author.

## Supplementary material

Supplementary Table 1 Characteristics of the selected participants for the 4 groups of the validation run. Participants selected for the validation run by ELISA were age-gender-BMI-FCW-matched to avoid any bias from such parameters in the discovery run to drive the altered expression of candidate cytokines in the validation run. For statistical comparison, using graph pad prism, Kruskal-Wallis test followed by Dunn test was performed to assess significant differences between the groups. P-values< 0.05 were considered significant. KK(+), Kato Katz positive (*S. mansoni* eggs-positive); KK(-), Kato Katz negative (*S. mansoni* eggs-negative); US(+), Positive for ultrasonography (clearly defined hepatic fibrosis); US(-), Negative for ultrasonography (No hepatic fibrosis); BMI, Body Mass Index; PPF, Periportal fibrosis; SD, standard deviation; Freq, Frequency; n, number; PPF, Periportal fibrosis; NA, Not applicable.

Supplementary Figure 1 Principal component analysis based on the expression levels of 27 plasma cytokines between the four groups of the discovery run following luminex assay. 4 phenotypic groups of patients, i.e., KK-US-, KK-US+, KK+US- and KK+US+. KK, Kato Katz; US, Ultrasonographic examination; (+), Positives; (-), Negatives.

Supplementary Figure 2 Discovery run Luminex results: All individual bar graphs comparing plasma cytokine expression levels between groups from the Luminex assay after the discovery run. Multiple comparisons of the levels of 27 plasma cytokines between the four groups of the discovery run following luminex assay. For statistical comparison, using graph pad prism, Kruskal-Wallis test followed by Dunn test was performed to assess significant differences between the groups. P-values< 0.05 were considered significant. Significant and suggestive cytokines are highlighted in grey (VEGF, p=0.1; GM-CSF, p=0.01; IL-2, p=0.03) and were further screened in downstream validation assays. Phenotypic groups of patients, i.e., KK-US-, KK-US+, KK+US- and KK+US+. KK, Kato Katz; US, Ultrasonographic examination; (+), Positives; (-), Negatives.

Supplementary Figure 3 Polyparasitism run Process used to select samples for the assessment of the impact of polyparasitism on plasma GM-CSF levels in our study population. Included participants were first screened for Schistosomiasis and geohelminth infections using the Kato Katz (KK) method, KK, Kato Katz; (+), Positives; (-), Negatives. SM, *Schistosoma mansoni*; GH, Geohelminths; HP, Hepatitis B/C; MA, Malaria. Plasma samples from participants found to be mono-, bi-, tri- or poly-infected were selected to constitute the polyparasitism cohort then screened by GM-CSF-specific ELISA to assess any eventual impact of any of these coinfections on GM-CSF levels. **(B)**. Impact of coinfections on SM negative GM-CSF levels. **(C)**. Impact of coinfections on SM positive GM-CSF levels. Statistical testing was done using non-parametric tests i.e. Kruskal-Wallis test followed by Dunn test for **(B)** and Mann-Whitney U test for **(C)**.
