# Supplementary material for: Reduced plasma levels of GM-CSF is a common feature of Schistosoma mansoni-infected school-aged children
Source: Front Immunol. 2025 Feb 28;16:1474575. doi: 10.3389/fimmu.2025.1474575 (PMC11906694; doi:10.3389/fimmu.2025.1474575)
Supplement: Supplementary Table 1 — Characteristics of the selected participants for the 4 groups of the validation run. Participants selected for the validation run by ELISA were age-gender-BMI-FCW-matched to avoid any bias from such parameters in the discovery run to drive the altered expression of candidate cytokines in the validation run. For statistical comparison, using graph pad prism, Kruskal-Wallis test followed by Dunn test was performed to assess significant differences between the groups. P-values< 0.05 were considered significant. KK(+), Kato Katz positive (S. mansoni eggs-positive); KK(-), Kato Katz negative (S. mansoni eggs-negative); US(+), Positive for ultrasonography (clearly defined hepatic fibrosis); US(-), Negative for ultrasonography (No hepatic fibrosis); BMI, Body Mass Index; PPF, Periportal fibrosis; SD, standard deviation; Freq, Frequency; n, number; PPF, Periportal fibrosis; NA, Not applicable. [file Image4.pdf]

Study Participants	group 1 (n=20) (KK$^{(+)}$ US$^{(+)}$)	group 2 (n=20) (KK$^{(+)}$ US$^{(-)}$)	group 3 (n=20) (KK$^{(-)}$ US$^{(+)}$)	group 4 (n=20) (KK$^{(-)}$ US$^{(-)}$)	p-values
**Age in years:**					
**Mean (SD)**	11.10 (2.63)	11.30 (2.05)	10.15 (1.70)	10.25 (1.48)	0.17
**Median (range)**	10.00 (7 – 16)	10.00 (8 – 15)	10.00 (7 – 14)	10.00 (8 – 13)	
**Sex ratio**					
**(Male / Female)**	10/10	10/10	11/09	11/09	0.97
**BMI**					
**Mean (SD)**	16.36 (1.50)	16.40 (1.28)	15.64 (1.36)	15.997 (1.42)	0.27
**Length of residence (years)**					
**Mean (SD)**	6.22 (2.73)	5.00 (4.70)	8.5 (0.70)	6.33 (3.21)	0.67
**Freq Contact with water/day**					
**Mean (SD)**	2.20 (0.83)	2.30 (0.65)	2.25 (0.68)	1.92 (0.76)	0.60
**Eggs Burden**					
**Median (range)**	36.00 (12 - 204)	48.00 (12 - 312)	NA	NA	0.47
**PPF grades**					
**Possible PPF**	13	NA	00	NA	NA
**PPF**	07	NA	17	NA	NA
**Advanced PPF**	00	NA	03	NA	NA
