# Supplementary figures and images for: Reduced plasma levels of GM-CSF is a common feature of Schistosoma mansoni-infected school-aged children
Source: Front Immunol. 2025 Feb 28;16:1474575. doi: 10.3389/fimmu.2025.1474575 (PMC11906694; doi:10.3389/fimmu.2025.1474575)

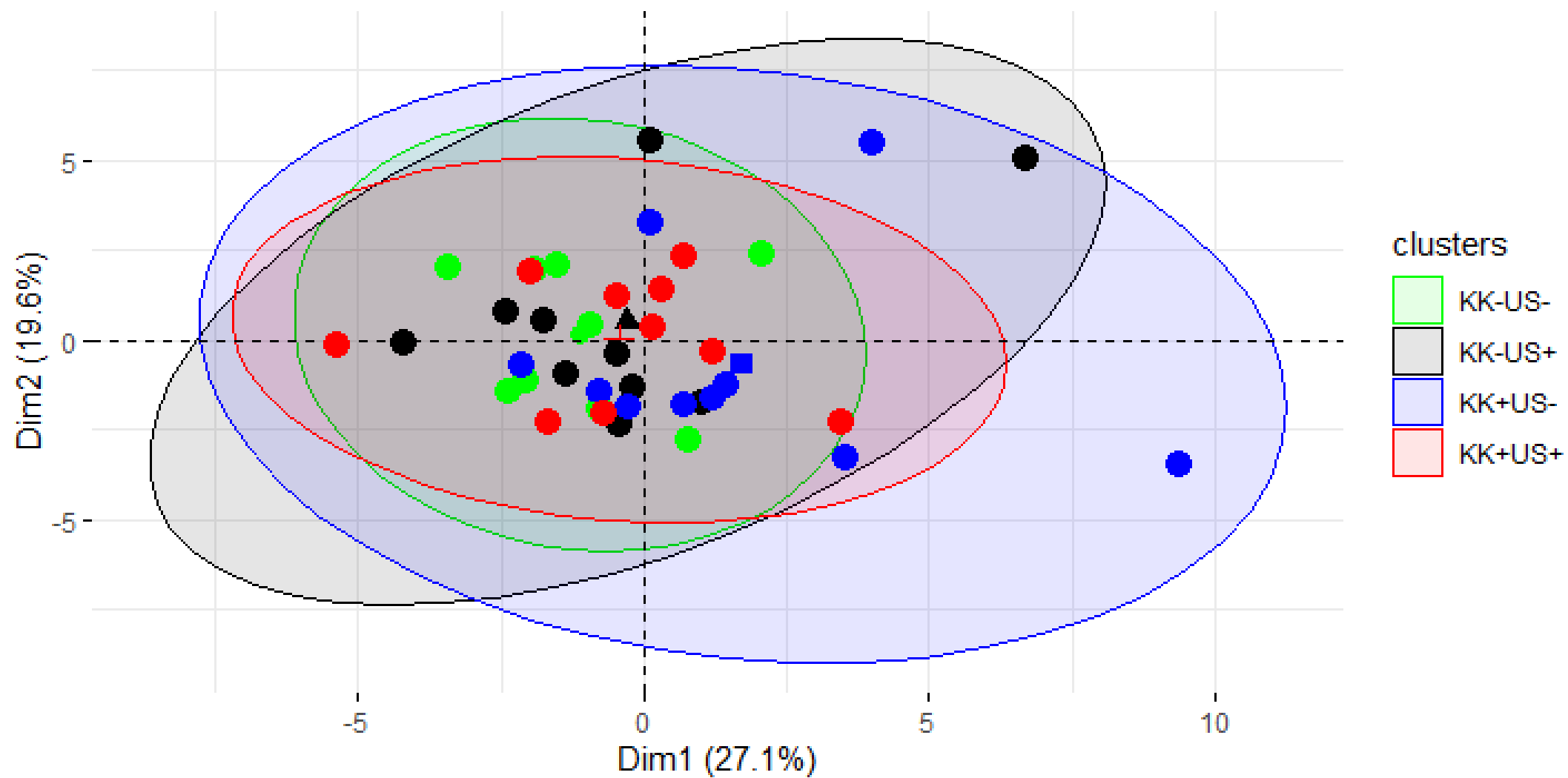

Supplement: Supplementary Figure 1 — Principal component analysis based on the expression levels of 27 plasma cytokines between the four groups of the discovery run following luminex assay. 4 phenotypic groups of patients, i.e., KK-US-, KK-US+, KK+US- and KK+US+. KK, Kato Katz; US, Ultrasonographic examination; (+), Positives; (-), Negatives. [file Image1.pdf]

A

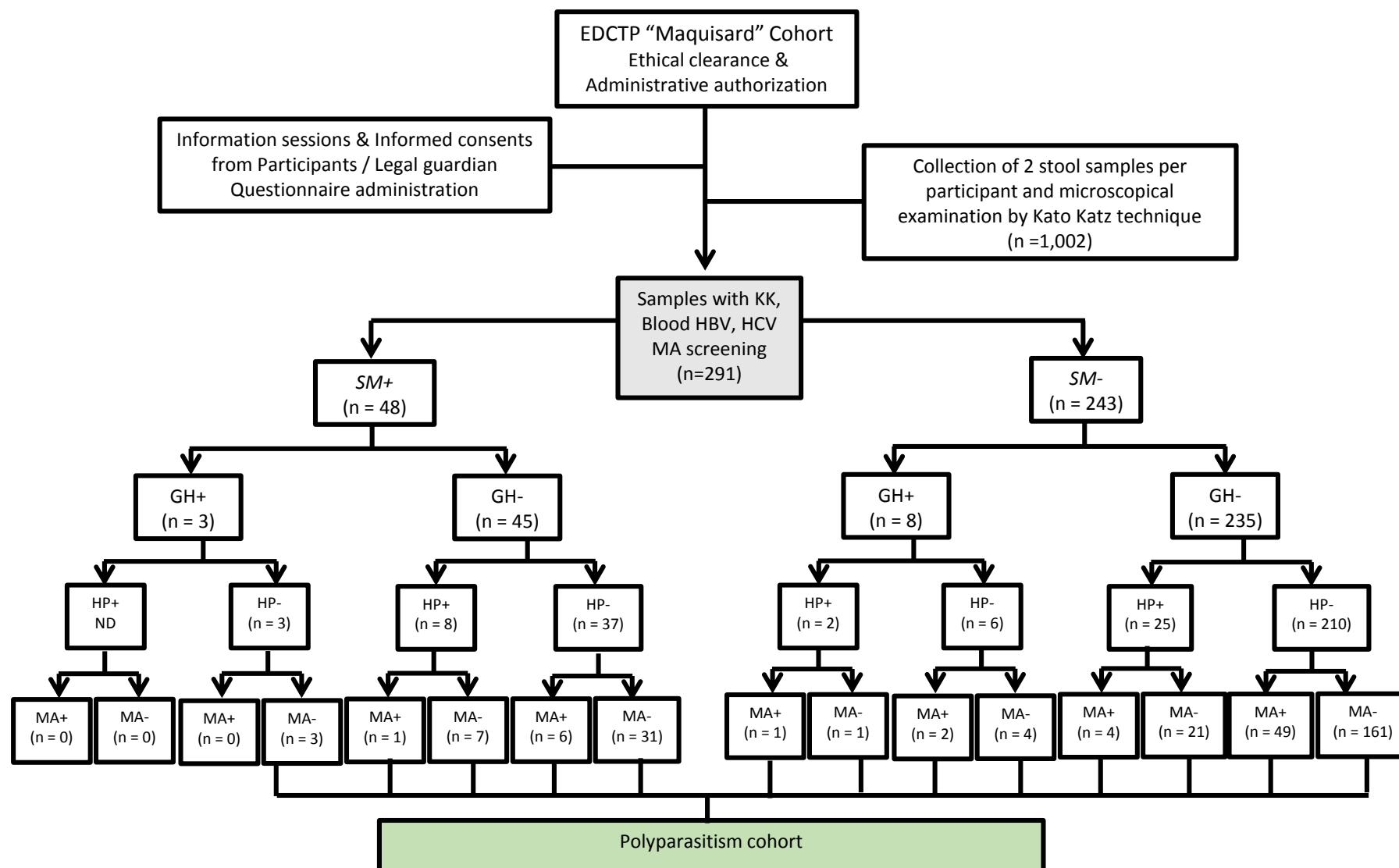

B

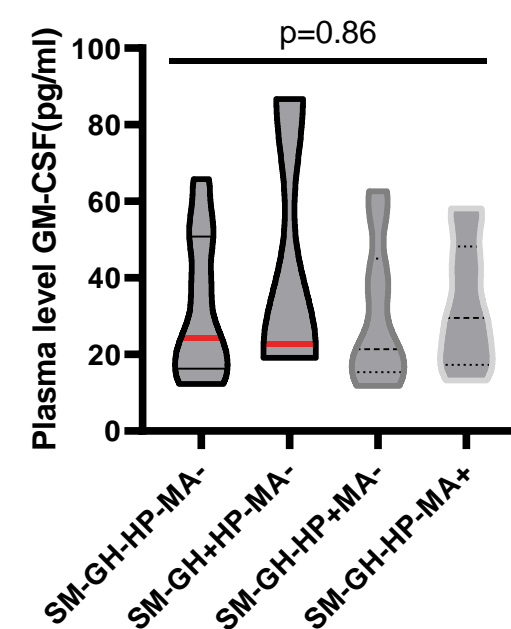

C

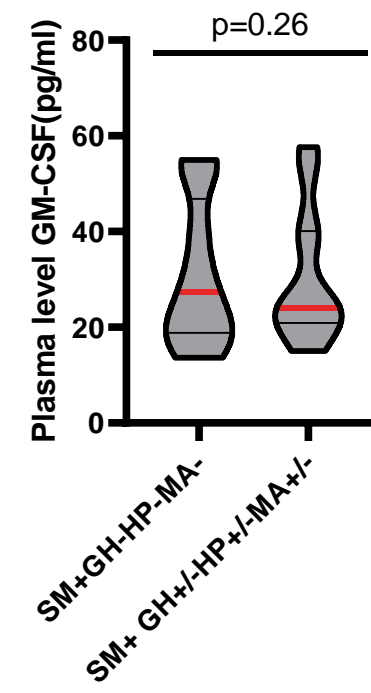

Supplement: Supplementary Figure 3 — Polyparasitism run Process used to select samples for the assessment of the impact of polyparasitism on plasma GM-CSF levels in our study population. Included participants were first screened for Schistosomiasis and geohelminth infections using the Kato Katz (KK) method, KK, Kato Katz; (+), Positives; (-), Negatives. SM, Schistosoma mansoni; GH, Geohelminths; HP, Hepatitis B/C; MA, Malaria. Plasma samples from participants found to be mono-, bi-, tri- or poly-infected were selected to constitute the polyparasitism cohort then screened by GM-CSF-specific ELISA to assess any eventual impact of any of these coinfections on GM-CSF levels. (B). Impact of coinfections on SM negative GM-CSF levels. (C). Impact of coinfections on SM positive GM-CSF levels. Statistical testing was done using non-parametric tests i.e. Kruskal-Wallis test followed by Dunn test for (B) and Mann-Whitney U test for (C). [file Image3.pdf]
