# Supplementary material for: Reduced plasma levels of GM-CSF is a common feature of Schistosoma mansoni-infected school-aged children
Source: Front Immunol. 2025 Feb 28;16:1474575. doi: 10.3389/fimmu.2025.1474575 (PMC11906694; doi:10.3389/fimmu.2025.1474575)
Supplement: Supplementary Figure 2 — Discovery run Luminex results: All individual bar graphs comparing plasma cytokine expression levels between groups from the Luminex assay after the discovery run. Multiple comparisons of the levels of 27 plasma cytokines between the four groups of the discovery run following luminex assay. For statistical comparison, using graph pad prism, Kruskal-Wallis test followed by Dunn test was performed to assess significant differences between the groups. P-values< 0.05 were considered significant. Significant and suggestive cytokines are highlighted in grey (VEGF, p=0.1; GM-CSF, p=0.01; IL-2, p=0.03) and were further screened in downstream validation assays. Phenotypic groups of patients, i.e., KK-US-, KK-US+, KK+US- and KK+US+. KK, Kato Katz; US, Ultrasonographic examination; (+), Positives; (-), Negatives. [file Image2.pdf]

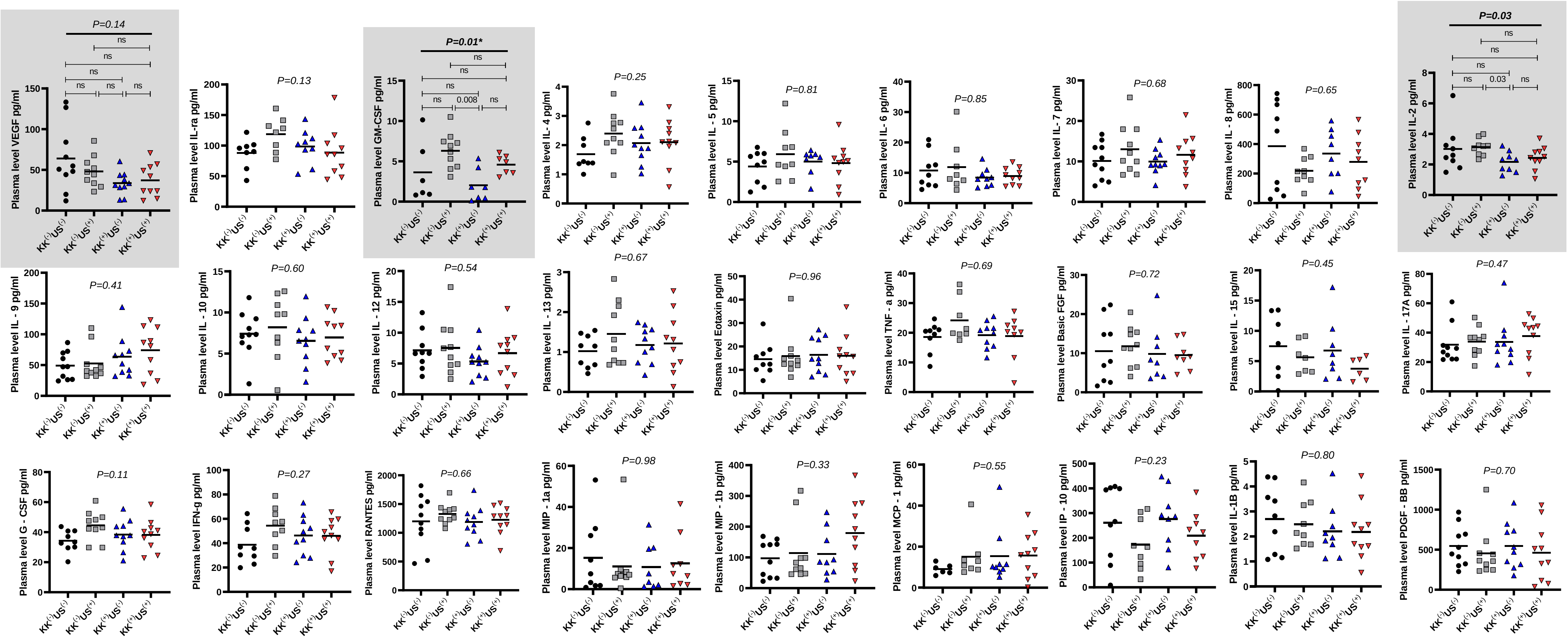

Plasma level VEGF pg/ml
P=0.14
Plasma level IL-ra pg/ml
P=0.13
Plasma level GM-CSF pg/ml
P=0.01*
0.008
Plasma level IL- 4 pg/ml
P=0.25
Plasma level IL- 5 pg/ml
P=0.81
Plasma level IL- 6 pg/ml
P=0.85
Plasma level IL- 7 pg/ml
P=0.68
Plasma level IL - 8 pg/ml
P=0.65
Plasma level IL-2 pg/ml
P=0.03
0.03
ns
Plasma level IL - 9 pg/ml
P=0.41
Plasma level IL - 10 pg/ml
P=0.60
Plasma level IL - 12 pg/ml
P=0.54
Plasma level IL -13 pg/ml
P=0.67
Plasma level Eotaxin pg/ml
P=0.96
Plasma level TNF - a pg/ml
P=0.69
Plasma level Basic FGF pg/ml
P=0.72
Plasma level IL - 15 pg/ml
P=0.45
Plasma level IL - 17A pg/ml
P=0.47
Plasma level G - CSF pg/ml
P=0.11
Plasma level IFN-g pg/ml
P=0.27
Plasma level RANTES pg/ml
P=0.66
Plasma level MIP - 1a pg/ml
P=0.98
Plasma level MIP - 1b pg/ml
P=0.33
Plasma level MCP - 1 pg/ml
P=0.55
Plasma level IP - 10 pg/ml
P=0.23
Plasma level IL-1B pg/ml
P=0.80
Plasma level PDGF - BB pg/ml
P=0.70
KK(-)US(-)
KK(-)US(+)
KK(+)US(-)
KK(+)US(+)
